# The complete chloroplast genome of *Rosa willmottiae* Hemsl. (1907)

**DOI:** 10.1080/23802359.2026.2742609

**Published:** 2026-10-07

**Authors:** Hui Zhang, Enguang Zhao, Dong Han, Jianqi Li, Ying Zhang, Jing Fang, Yuan Niu, Xing He, Lingling Da, Chaodong Hu

**Affiliations:** ^a^ College of Life Science, Northwest Normal University, Lanzhou, China; ^b^ Lanzhou Agro-Technical Research and Popularization Center, Lanzhou, China; ^c^ Lanzhou Academy of Agricultural Sciences, Lanzhou, China

**Keywords:** *Rosa willmottiae*, *Rosa*, Kushui Rose, chloroplast genome, phylogenetic analysis

## Abstract

*Rosa willmottiae* Hemsl. (1907) is an ornamental plant with certain medicinal value. Its complete chloroplast genome was assembled via high-throughput sequencing. The 157,277 bp circular genome comprises one large single-copy region (LSC; 86,207 bp), one small single-copy region (SSC; 19,264 bp) and two inverted repeat regions (IRs; 25,903 bp each), encoding 130 genes: 86 protein-coding genes, 36 tRNAs and 8 rRNAs. Phylogenetic analyses indicate *Rosa willmottiae*’s closest affinity to Kushui Rose (*R. sertata × R. rugosa*), and strongly support *Rosa willmottiae*’s role as the putative maternal progenitor of Kushui Rose. This study contributes to the understanding of evolutionary relationships within the genus *Rosa*.

## Introduction

Roses have been renowned for their beautiful flowers and pleasant fragrance, possessing immeasurable aesthetic value as ornamental plants in gardens. *R. willmottiae* Hemsl. (1907) is a shrub species of the genus *Rosa* in the family Rosaceae. Similar to other fragrant roses, volatile aromatic compounds in its petals impart a pleasant scent (Wang 2019). While having high horticultural value, it also possesses medicinal value. Phytochemical studies have shown that its flowers are rich in terpenoids and phenolic compounds, which serve as the main material basis for its medicinal efficacy. Its flowers can be used to treat fever, diarrhea, menstrual disorders, and other conditions (Ye 2016).

Chloroplast genome sequencing holds significant potential for the analysis of phylogenetic relationships, genetic diversity, and biological evolution (Shinozaki et al. 1986). Within the genus *Rosa*, chloroplast genomes have been increasingly employed to clarify species delimitation, hybridization events, and maternal lineages, offering insights into the complex evolutionary patterns of this taxonomically challenging group. Studies on *Rosa* chloroplast genomes have revealed conserved structures and moderate variation, providing valuable resources for DNA barcoding and comparative genomics. Therefore, we sequenced the complete chloroplast genome of *R. willmottiae* using Illumina, conducted comprehensive assembly and annotation, and provided insights into its phylogenetic position within the genus *Rosa*.

## Materials and methods

### DNA extraction, genome sequencing and raw data quality control

Fresh and well-grown leaves of *R. willmottiae* were collected on 20 August 2023 from the plantation of the College of Life Sciences, Northwest Normal University (36.098131˚N, 103.738272˚E), Gansu, China, and their total mixed genomic DNA was extracted by the CTAB method. A specimen was deposited at the Institute of Botany, College of Life Science, Northwest Normal University (https://sky.nwnu.edu.cn; Dong Han, 372965350@qq.com) under the voucher number RW231012. The quality and integrity of the DNA were examined by agarose gel electrophoresis and the Nanodrop method. According to the manufacturer’s instructions (Illumina, USA), DNA libraries with different indices were multiplexed and loaded on an Illumina NovaSeq 6000 platform. The libraries were sequenced using a 2 × 150 paired-end sequencing strategy. Sequencing quality and base composition of each site were assessed using FastQC (v.0.11.8) (Andrews 2010). Additionally, seqtk (v.1.3-r106) was used to assess the base quality and sequencing error rate. Then, fastp (v.0.19.5) (Chen et al. 2018) was used to remove adapter sequences and low-quality sequences, and Kraken2 (v.2.0.9) (Wood et al. 2019) was used to analyze and identify chloroplast genome sequences in the sequencing data. Finally, data statistics and mapping were performed using R (v.3.6.1) (R Core Team 2019).

### Chloroplast genome assembly

Chloroplast genome assembly was performed using metaSPAdes (v.3.13.0) (Nurk et al. 2017) to assemble the sequencing reads into contigs. The complete chloroplast genome sequence of *R. sertata* (GenBank: NC_066975) was used as a reference to validate the assembly. This step helped determine whether the chloroplast genome formed a circular structure, verify contig orientation, and identify the start base position. The assembled chloroplast genome sequence of *R. willmottiae* was submitted to NCBI under GenBank accession number PP577899.

### Chloroplast gene annotation

CPGAVAS2 (Shi et al. 2019) was used to annotate the chloroplast genome of *R. willmottiae*, while the circular chloroplast genome was visualized using OGDRAW (v.1.1.1) (Lohse et al. 2013).

### Analysis of long tandem repeats and SSRs

SSRs were analyzed using MISA (Beier et al. 2017) with search parameters of ten repeat units for mononucleotide SSRs, five repeat units for dinucleotide SSRs, four repeat units for trinucleotide SSRs, and three repeat units for tetranucleotide, pentanucleotide, and hexanucleotide SSRs. Additionally, long tandem repeats were detected with Tandem Repeats Finder (Benson 1999) using default parameters.

### Calculate relative synonymous codon usage (RSCU)

Relative synonymous codon usage (RSCU) for protein-coding genes was calculated using the RSCU module in the CAI Python package (v.1.0.3) (Sharp and Li 1987).

### Phylogenetic position analysis

Phylogenetic trees were constructed with the chloroplast genome of *R. willmottiae* generated in this study and complete chloroplast genomes of 47 additional species from NCBI, with *Arabidopsis thaliana* (GenBank: NC_000932.1) selected as the outgroup for tree rooting. All sequences were aligned using MAFFT (v.7.520) (Katoh and Standley 2013) with default parameters, followed by trimming with trimAl (v.1.2) (Capella-Gutiérrez et al. 2009). The final phylogenetic tree was inferred using IQ-TREE (v2.2.6) (Minh et al. 2020) with the maximum likelihood (ML) method; the optimal nucleotide substitution model was automatically selected by ModelFinder, and branch support was assessed with 1000 bootstrap replicates, while other parameters remained default.

## Results

### Characteristics of the chloroplast genome of *R. willmottiae*

Raw chloroplast genome data of *R. willmottiae* were obtained by Illumina sequencing, which yielded 24,697,620 reads (3,693,392,809 bp). After quality control, Q20 and Q30 values were 96.8% and 91.9%. The complete chloroplast genome of *R. willmottiae* (Figure 1) is a typical circular double-stranded DNA molecule of 157,277 bp, comprising four regions: a large single-copy (LSC) region (86,207 bp), a small single-copy (SSC) region (19,264 bp), and two inverted repeat (IR) regions (25,903 bp each). The sequencing coverage depth across the genome assembly was assessed, with an average sequencing depth of 2077× (Supplementary Figure S1). The overall GC content was 37.2%. Regionally, IR regions exhibit significantly higher GC content (42.8%) than the LSC (35.2%) and SSC (31.3%) regions. The structures of cis- and trans-splicing genes were illustrated in Supplementary Figure S2.

**Figure 1.**
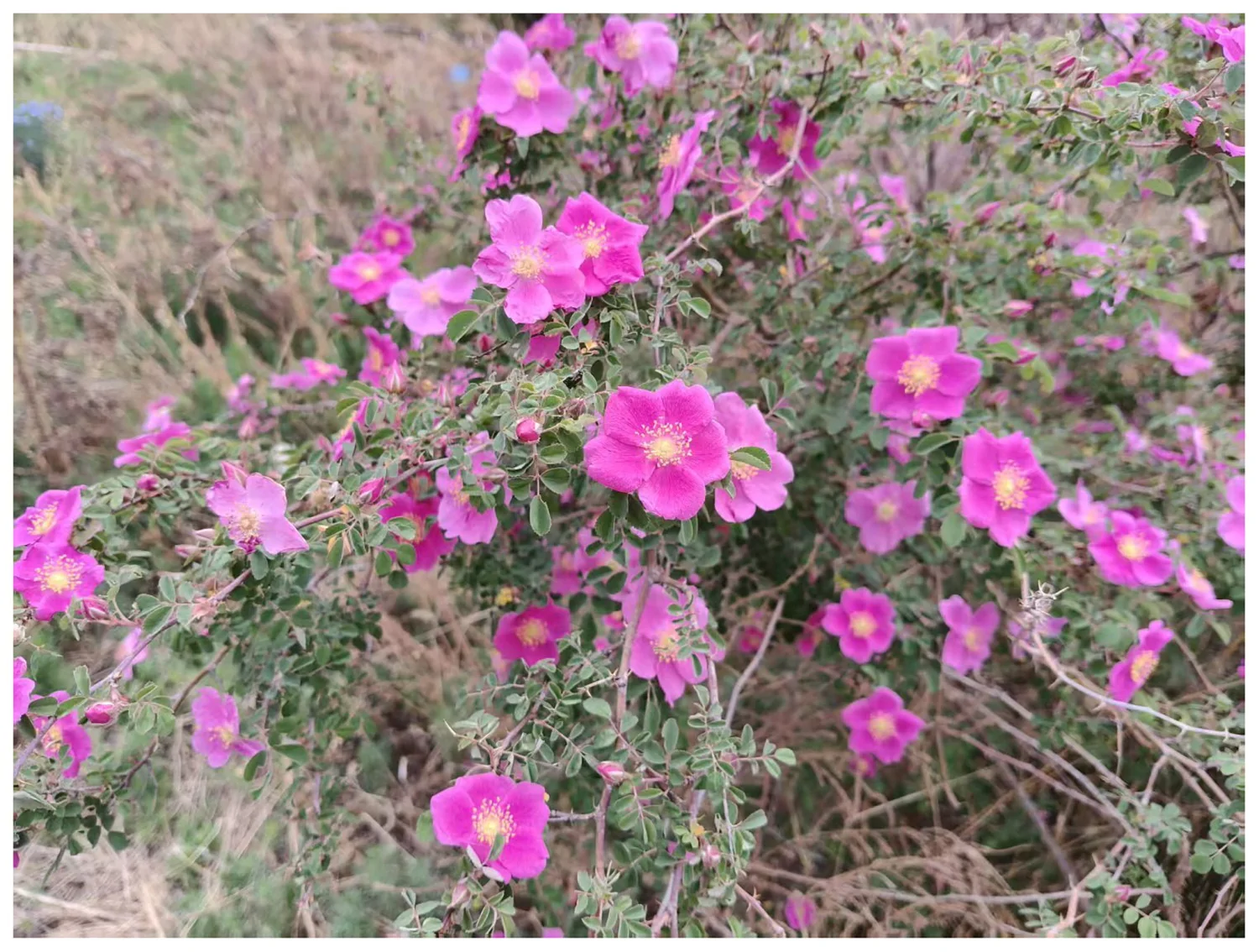
The species image of *R. willmottiae*. The flowers of *R. willmottiae* are small, pink to lilac-purple, with a simple, open form of five petals and a prominent central cluster of yellow stamens. The photograph was taken by Jianqi Li at the plantation of the College of Life Sciences, Northwest Normal University, Gansu, China.

The chloroplast genome of *R. willmottiae* encodes 130 genes, including 86 protein-coding genes, 36 tRNA genes and 8 rRNA genes (Figure 2). 18 genes are located in both IR regions, comprising six protein-coding genes (*rps12, rps7, ndhB, ycf2, rpl23, rpl2*), 4 rRNA genes (*rrn5, rrn4.5, rrn23, rrn16*) and 8 tRNA genes (*trnN-GUU, trnR-ACG, trnA-UGC, trnS-GCU, trnI-GAU, trnV-GAC, trnL-CAA, trnI-CAU*). The SSC region harbored 10 protein-coding genes and 1 tRNA gene. The LSC region includes 63 protein-coding genes and 19 tRNA genes. 106 genes consist of single exons, while 22 genes contain one intron and 2 genes contain two introns (Supplementary Table S1).

**Figure 2.**
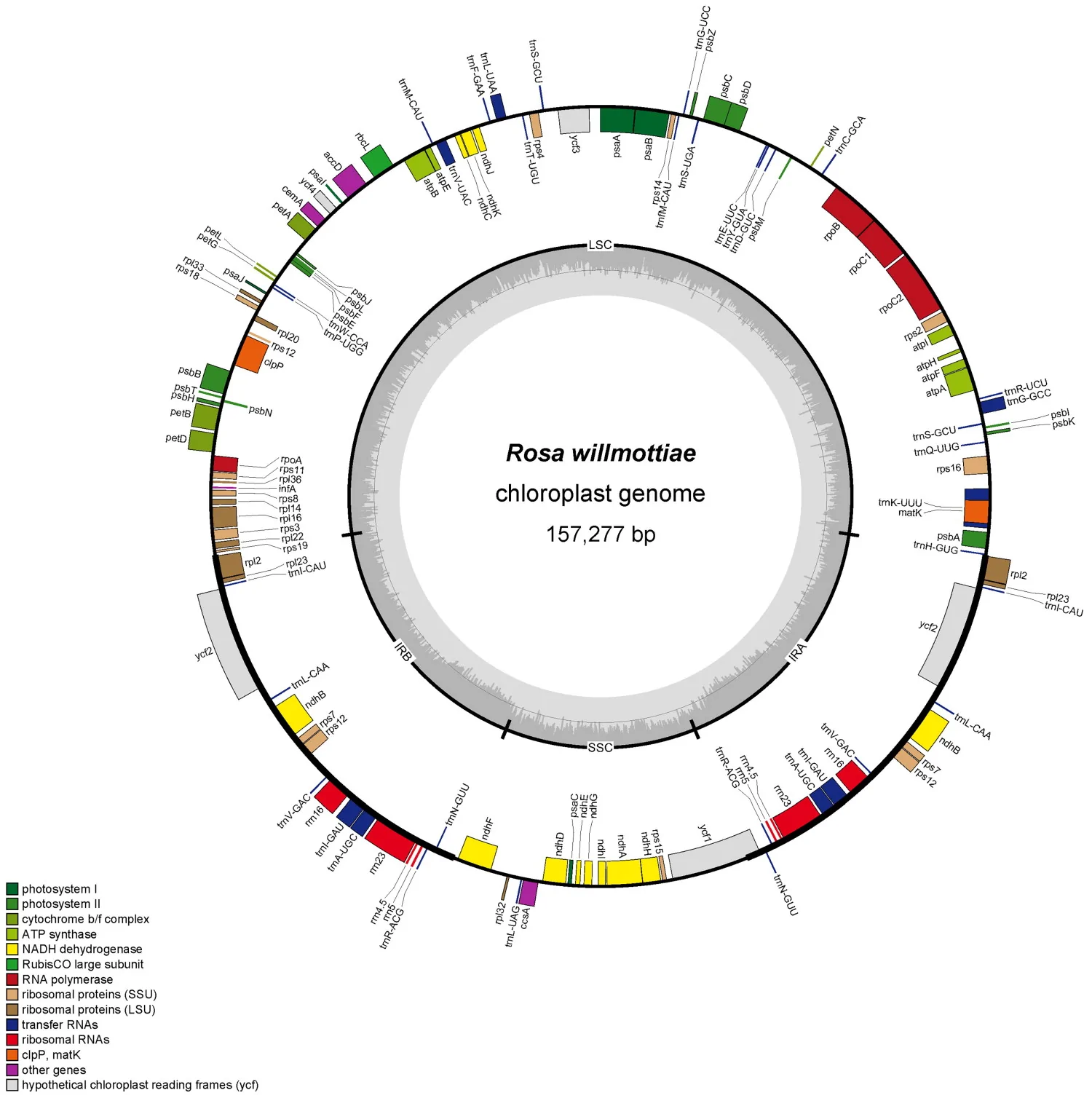
The circular map depicts the chloroplast genome of *R. willmottiae*. Genes drawn inside the circle are transcribed clockwise, while those outsides are transcribed counterclockwise. Genes are color-coded to imply functional groups. The inner circle displays GC content (dark gray) and AT content (light gray). The SSC, LSC, IRa, and IRb regions are labeled within the inner circle. The *rps12* is a trans-spliced gene with the 5′end located in the LSC region and duplicated in the 3′ end in the IR regions.

### SSRs and long tandem repeats

In the *R. willmottiae* chloroplast genome, we detected 84 SSRs (Supplementary Table S2), including 50 mononucleotide repeats. Among these mononucleotide repeats, 46 consisted exclusively of A or T bases. Of the remaining 34 SSRs, over 50% contained A/T bases. This AT-rich SSR composition aligns with the overall AT bias of the chloroplast genome. Additionally, 47 long tandem repeats were identified (Supplementary Table S3).

### Codon usage analysis

We detected 26,044 codons across all genes (Supplementary Table S4). Leucine was the most abundant amino acid (2,742; 10.5%), followed by isoleucine (2,242; 8.6%), while cysteine was the least abundant (304; 1.2%). 29 codons exhibited RSCU values >1.00, with TTA showing the highest value (1.912). The lowest RSCU value is GAC (0.378).

### Analysis of phylogenetic relationships

To clarify the phylogenetic position of *R. willmottiae* within the genus Rosa, chloroplast genome data from 48 species were selected, a maximum likelihood (ML) phylogenetic tree was constructed, and nodal support was assessed with 1,000 bootstrap replicates (Figure 3 and Supplementary Figure S3). In the phylogenetic tree, all Rosaceae species were divided into three groups. *R. willmottiae* belongs to Sect. *Cinnamomeae* in group 1 together with *R. rugosa*, *R. sertata*, Kushui Rose (*R. sertata* × *R. rugosa*) and *R. davurica*, *R. willmottiae* was phylogenetically closest to Kushui Rose, and the two species belonged to the same subclade with a bootstrap support value of 99.

**Figure 3.**
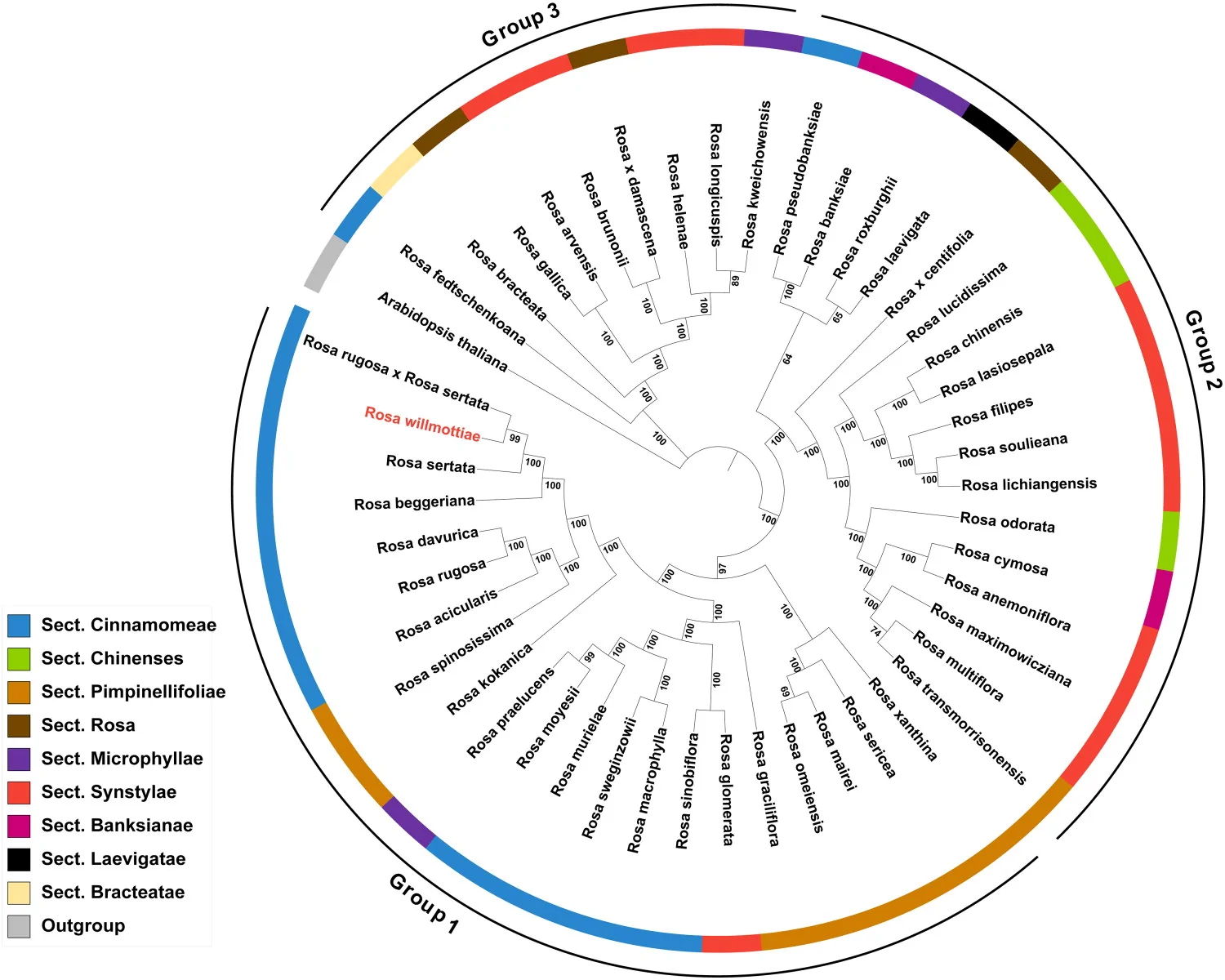
The phylogenetic tree was constructed using the maximum likelihood (ML) method based on the alignment of complete chloroplast genome sequences of *R. willmottiae* and 47 other representative species, with *Arabidopsis thaliana* as the outgroup. Numbers above the branches indicate bootstrap support values derived from 1000 replicates. The species under study is displayed in red font. The following chloroplast genome sequences were used: *R. willmottiae*, PP577899.1; *R. acicularis*, OP032238.1 (Gao et al. 2023); *R. anemoniflora*, NC_083977.1 (Gao et al. 2024); *R. arvensis*, NC_061263.1 (Cui et al. 2022); *R. banksiae*, NC_042194.1 (Wang et al. 2019); *R. beggeriana*, NC_079961.1; *R. bracteata*, NC_061270.1 (Cui et al. 2022); *R. brunonii*, NC_061267.1 (Cui et al. 2022); *R. chinensis*, NC_065867.1; *R. cymosa*, NC_051550.1 (Ding et al. 2020); *R. davurica*, NC_057236.1; *R. fedtschenkoana*, NC_061268.1 (Cui et al. 2022); *R. filipes*, NC_053856.1 (Wang et al. 2020); *R. gallica*, NC_061271.1 (Cui et al. 2022); *R. glomerata*, NC_062462.1 (Chen et al. 2022); *R. graciliflora*, OQ992658.1; *R. helenae*, NC_061982.1 (Cui et al. 2022); *R. kokanica*, MW298478.1 (Choi et al. 2021); *R. kweichowensis*, NC_061983.1 (Cui et al. 2022); *R. laevigata*, NC_046824.1 (Zhang et al. 2019); *R. lasiosepala*, NC_071930.1; *R. lichiangensis*, NC_079928.1; *R. longicuspis*, NC_061987.1 (Cui et al. 2022); *R. lucidissima*, MK782979.1 (Zhao et al. 2019); *R. macrophylla*, OQ992659.1; *R. mairei*, NC_079931.1; *R. maximowicziana*, NC_040960.1 (Jeon and Kim 2019); *R. moyesii*, NC_079932.1; *R. multiflora*, NC_039989.1 (Zhang et al. 2022); *R. murielae*, NC_079933.1; *R. odorata*, KF753637.1 (Yang et al. 2014); *R. omeiensis*, OQ992648.1; *R. praelucens*, NC_037492.1 (Jian et al. 2018); *R. pseudobanksiae*, NC_079869.1; *R. roxburghii*, NC_032038.1 (Wang et al. 2018); *R. rugosa*, NC_044094.1 (Kim et al. 2019); *R. rugosa × R. sertata*, NC_062657.1 (Niu et al. 2022); *R. sericea*, OQ992650.1; *R. sertata*, NC_066975.1; *R. sinobiflora*, NC_079870.1; *R. soulieana*, NC_079871.1; *R. spinosissima*, NC_079872.1; *R. sweginzowii*, NC_080343.1; *R. transmorrisonensis*, NC_065110.1; *R. x centifolia*, NC_079962.1; *R. x damascena*, MZ261851.1 (Cui et al. 2022); *R. xanthina*, NC_051543.1 (Gao et al. 2020); *A. thaliana*, NC_000932.1 (Sato et al. 1999).

## Discussion and conclusion

In this study, we assembled the complete chloroplast genome of *R. willmottiae* (157,277 bp), containing 86 protein-coding genes, 36 tRNAs, and 8 rRNAs. Within these genes, 26,044 codons were identified. We detected 84 SSRs in *R. willmottiae*, which can serve as molecular genetic markers for differentiating closely related species.

It has long been recognized that the Kushui Rose is a natural hybrid between *R. rugosa* and *R. sertata* (Ma et al. 1985). However, a study combining morphological trait identification, nuclear DNA analysis (ITS and GAPDH), and chloroplast gene trees concluded that Kushui Rose originated from *R. rugosa* and *R. willmottiae*, not *R. sertata* (Zhao et al. 2024). Our phylogenetic tree shows that *R. willmottiae* and Kushui Rose occupy closer phylogenetic positions than *R. sertata* and Kushui Rose, consistent with the above report.

In summary, this study provides the complete chloroplast genome data of *R. willmottiae* and offers new insights into its phylogenetic relationships, which may facilitate the further development and utilization of this plant resource. Future studies should integrate population genomics and metabolomics approaches to further elucidate the genetic basis and adaptive evolutionary mechanisms underlying their phylogenetic affinity.

## Supplementary Material

Supplemental Material

Supplemental Material

## Data Availability

The genome sequence data that support the findings of this study are openly available in GenBank of NCBI at [https://www.ncbi.nlm.nih.gov] under the accession PP577899. The associated BioProject, SRA, and Bio-Sample numbers are PRJNA1307760, SRR35030388, and SAMN50672542 respectively.
