# Supplementary material for: The complete chloroplast genome of Rosa willmottiae Hemsl. (1907)
Source: Mitochondrial DNA B Resour. 2026 Oct 7;11(11):1272–7. doi: 10.1080/23802359.2026.2742609 (PMC13647347; doi:10.1080/23802359.2026.2742609)
Supplement: Supplementary file 1 [file TMDN_A_2742609_SM1365.docx]

Figure S1 Coverage Depth Across the *R. willmottiae* Genome Assembly. The X-axis represents the continuous base positions along the genome, and the Y-axis represents the sequencing coverage depth. The coverage depth, measured in reads per base (×), varies across different genomic regions, with a genome-wide average sequencing depth of 2077×, a minimum depth of 103× and a maximum depth of 6201×. The total span of the assembly shown is 157,277 base pairs.

Figure S2 Schematic diagrams of cis-splicing genes (A) and the trans-splicing gene *rps12* (B) in the chloroplast genome of *R. willmottiae.*

(A) Exons and introns are shown in black and white, respectively. Arrows indicate the sense direction of transcription, and the numbers below each schematic represent the genomic coordinates of the corresponding sequences in the chloroplast genome. (B) Arrows on exons indicate the sense direction of transcription, and the numbers below each schematic denote the genomic positions of the corresponding sequences in the chloroplast genome.

Figure S3 The phylogram was constructed using the maximum likelihood (ML) method based on the alignment of complete chloroplast genome sequences from *R. willmottiae* and 47 other representative species. Numbers above the branches indicate bootstrap support values derived from 1000 replicates.
